# Improving water competency among children on the autism spectrum: the AquOTic randomized controlled trial

**DOI:** 10.3389/fped.2024.1473328

**Published:** 2024-10-07

**Authors:** Erika Kemp, Melica Nikahd, Mequeil Howard, Amy Darragh, Jewel E. Crasta

**Affiliations:** ^1^ Occupational Therapy Division, The Ohio State University, Columbus, OH, United States; ^2^ Center for Biostatistics, The Ohio State University, Columbus, OH, United States

**Keywords:** autism, swim skills, AquOTic, water competency, intervention, occupational therapy

## Abstract

**Introduction:**

There is a critical need for evidence-based and manualized interventions targeting water competency including swim and water safety skills tailored to meet the needs of children on the autism spectrum, a group that is at a high risk of drowning. This study examined the efficacy of AquOTic—a 10-week occupational therapy-based aquatic intervention to improve water competency among children on the autism spectrum.

**Methods:**

A total of 37 children on the autism spectrum (ages 5–9 years) were randomized to a waitlist control group (*n* = 24) or AquOTic intervention group (*n* = 37; 28 males). Blinded assessors administered the standardized Water Orientation Test-Alyn (WOTA) 1 and 2 and a Swim Skills Checklist to all participants pre- and post-AquOTic/control. Repeated measures mixed effects models were used to examine intervention effects.

**Results:**

Average WOTA 1 scores increased significantly after participants received AquOTic (Δ = 5.7; 95% CI: 3.7–7.8; *p* < 0.001), and average WOTA 2 scores increased significantly after participants received AquOTic (Δ = 9.0; 95% CI: 5.7–12.3; *p* < 0.001). Average swim skills increased significantly after participants received AquOTic (Δ = 7.6; 95% CI: 5.3, 10.0; *p* < 0.001).

**Conclusion:**

Our results highlight the efficacy of AquOTic to improve water competency among children on the autism spectrum. Further research is needed to examine long-term effects, dosage requirements to achieve water competency, and the impact of aquatic therapy on other health outcomes.

**Clinical Trials Registration:**

clinicaltrials.gov, NCT05524753.

## 1 Introduction

Autism Spectrum Condition (henceforth “autism”) is a neurodevelopmental condition, associated with social, communication, and behavioral differences, with an estimated prevalence of 1 in 36 children (1). Approximately one-third have a co-occurring intellectual disability (1) and up to 87% show motor differences (2). Drowning is a leading cause of unintentional injury death in children on the autism spectrum, accounting for about 90% of deaths in children ages 14 and younger (3). The wide array of sensory, cognitive, and motor challenges associated with autism often limit participation in traditional swim lessons (4). In addition, high rates of wandering and elopement in children on the autism spectrum are a primary contributing factor to fatal drowning (3). Over 70% of lethal elopement cases can be attributed to accidental drowning (5). Moreover, individuals in the autism spectrum are twice as likely to die from drowning than the general population (6). Thus, there is a critical need for therapeutic water competency interventions for children on the autism spectrum. Water competency includes skills of entering the water safely, getting a breath, staying afloat, changing positions, swimming a distance, and then getting out of the water (7). Should a child fall or wander into a pool, lake, or other bodies of water, these are the essential skills for returning to land safely. Additional skills that can increase the chance of drowning survival include knowing what the sound of a whistle means, knowing who lifeguards are and what their job is, recognizing a person in distress, knowing when to call for help, and how to safely provide flotation to others (8). Gaining proficiency in water competency is a protracted process requiring multiple instructional sessions combining learning alongside developmental maturation (8).

Formal swim instruction in young neurotypical children significantly reduces the risk of drowning (9). However, traditional methods of instruction may be of limited benefit for children on the autism spectrum due to differences in learning, sensory, motor, behavioral, and social skills (10). Traditional swim lessons are designed for neurotypical children, taught by one instructor to a group of children using a set curriculum, and instructors have very little information, if any, on how to adapt lessons for children with different learning needs. Parents consistently report that their children on the autism spectrum do not learn to successfully swim in traditional swim lessons (4, 10–12). Despite the risk, swimming has been ranked as one of the most enjoyed activities for children on the autism spectrum (13). Currently, no evidence-based form of water competency and safety exists as the standard of care for these children.

There is growing consensus that swim instruction is effective in improving water competency in children on the autism spectrum (14–16). Synthesis of existing swim interventions for children on the autism spectrum shows that programs offer a multifaceted approach targeting physical, sensory, and social skills. Most autism aquatic intervention programs are tailored to the child's individual needs, incorporating structured, sensory-friendly practices and involving family support. The Halliwick method is a widely used instruction approach, comprised of a 10-point program that uses the hydrostatic and hydrodynamic water properties to progressively teach swim skills from easy to complex movements and is usually taught within group settings (17–19). A recent review (14) showed that the majority of programs (12 out of 19 studies analyzed) were offered 60 min per week, with program durations ranging from 30 to 90 min. The instructor/therapist child supervision ratio was 1:1 in 13 out of the 19 studies analyzed, with most programs being led by occupational or physical therapists or certified swim instructors (14). Duration varied from 4 h to 72 h (over 10 months). Significant improvements in swim skills have been shown at a minimum of 10 h of intervention (22, 26) with continued improvements through 28 h of intervention (24). A recent meta-analysis showed that aquatic programs significantly improved motor and social skills among children on the autism spectrum (14). In addition to gaining life-saving swim skills, increasing swim abilities also leads to better overall health outcomes (4, 15, 17, 19–21).

Despite growing evidence supporting swim interventions in autism, there are critical gaps in the literature and the need for rigorous intervention trials. Recent systematic reviews (14, 15, 21, 22) of swim interventions for children on the autism spectrum showed that more than half contained sample sizes of less than 7 participants, had significant methodological concerns, lacked control groups, did not use standardized measures, or followed a manualized evidence-based intervention. They concluded that high-quality randomized controlled trials (RTCs) of swim and water competency were needed.

In this article, we present results from an RCT of an occupational therapy-based water competency intervention—*AquOTic* among children on the autism spectrum. AquOTic is a manualized 10-week swim intervention that includes evidence-based techniques such as play-based and child-led activities, task-specific training, positive reinforcement, sensory supports, and a modified Halliwick approach to swim instruction (23). Our prior feasibility study of AquOTic among eight children on the autism spectrum showed that AquOTic was safe with no adverse events and feasible as measured by high intervention fidelity and low attrition (23). AquOTic also had high caregiver satisfaction and child participants showed significant improvements in swim skills. Expanding on our earlier work, in the current study, we examined the efficacy of AquOTic to improve water competency skills among children on the autism spectrum using a single-blind RCT design. We hypothesized that children receiving the AquOTic intervention would show greater improvements in standardized measures of swim skills and water competency criteria compared to children in the waitlist control group.

## 2 Methods

This study was conducted at The Ohio State University and registered at clinicaltrials.gov (Clinical Trials NCT05524753). Study procedures were approved by the local university's Institutional Review Board. All families provided signed informed consent.

### 2.1 Participants

Participants (*n* = 37) aged 5–9 years on the autism spectrum were recruited using a waitlist control design. Participants were randomized using a computer-generated randomization schedule to one of three groups, (1) the AquOTic intervention-first; (2) Waitlist control *a*; or 3. Waitlist control *b*. Participants randomized to the AquOTic intervention first (*n* = 12) began the AquOTic intervention immediately in fall 2022. Participants assigned to the waitlist control groups *a* and *b* (*n* = 25) served as controls for a period of at least 4 months and then were offered the opportunity to participate in the AquOTic intervention. Those assigned to waitlist control *a* received the AquOTic intervention during Spring 2023 while those in waitlist control b received the AquOTic intervention during Spring/Summer 2023. Children were recruited between July 2022 and September 2022 through the local County Board of Developmental Disabilities, social media posts, word-of-mouth, and flyers distributed throughout the local university. Participants were included if they: (1) had a confirmed diagnosis of autism by a healthcare provider, (2) were between the ages of 5–9 years, (3) had normal or corrected vision and hearing, (4) had intact airways, and (5) no uncontrolled seizures. Children who demonstrated swim proficiency, as defined by the ability to float for 5–10 s or move their body through the water without flotation on parent report, were excluded. As an incentive, families received $25 for each assessment session they attended and the AquOTic intervention sessions were provided at no charge with no additional incentives.

### 2.2 Procedures

**Figure 1:**
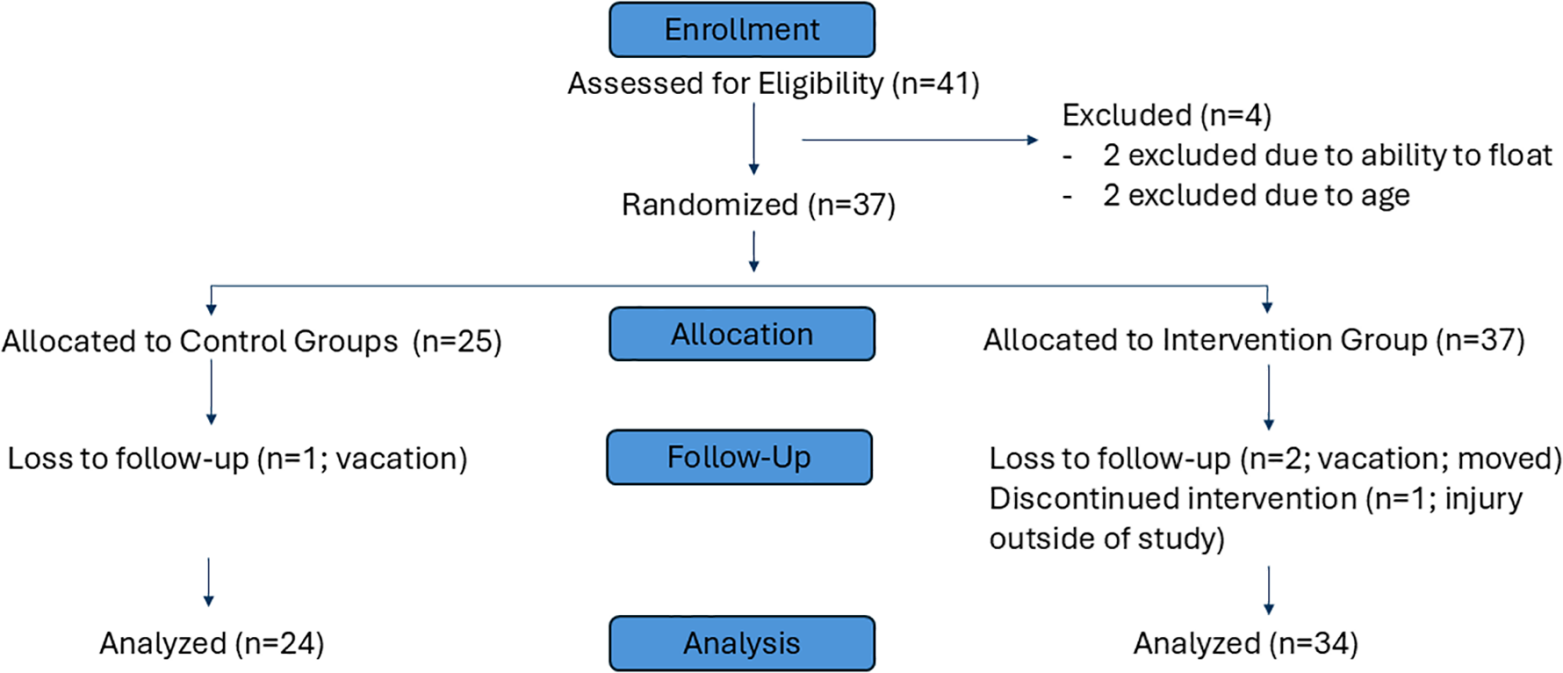
Consolidated standards for reporting trials (CONSORT) flow diagram.

Altogether, 41 participants were assessed for eligibility and 37 participants were randomized to AquOTic intervention or control groups see Figure 1. All participants completed a baseline and post-intervention/control assessment with a blinded trained assessor. Both the child participant and blinded assessor were in the water at the time of the assessments which typically occurred over 30 min.

#### 2.2.1 Aquotic intervention protocol

The AquOTic intervention is an occupational therapy-based water competency and safety intervention tailored for children on the autism spectrum (23). AquOTic provides individualized therapeutic swim intervention in a group setting, with one interventionist assigned to each child throughout the program. There are 6 child-interventionist dyads per session, supervised by a lead therapist. Six dyads represent best practice for therapeutic groups (24). Each child is paired with the same 1:1 interventionist, a current graduate-level therapy student, to develop a therapeutic relationship and provide individualized intervention. The program occurs once a week over 10 weeks in a warm water pool located at a local school for children with developmental disabilities. Each 60-min intervention session follows a structured routine with individualized activities. The interventionists individualize the intervention each week to each child's needs and goals. The baseline land and water-based assessment informs the creation of individualized goals for each child see Table 1.

**Table 1:** Description of AquOTic protocol and session structure.

Activities	Time	Location	Examples
Rules	5 min	Pool deck	“Use walking feet”, “Jump where it's clear”
Water adjustment	5 min	Edge of pool	Sponges, painting, play with interventionist
Skill introduction	5 min	In the pool	Bubbles, back float, kicking, etc.
Station rotations	35 min	In the pool	Rotate to 6: submerge, float, monkey crawl, rope pull, jumping, throw & scoop, kicking, treading, individual skills
Closing game/song	5 min	In the pool	Wheels on the bus, goodbye, etc.
Caregiver feedback	5 min	Pool deck	Skills learned/emphasized today. Week 10: caregiver in water
Total	60 min		

Each session begins with swim safety instruction including concepts such as using walking feet, who a lifeguard is, what to do when you hear a whistle, and where it is safe to jump into the pool. Water adjustment follows with a song for water acclimation, followed by 2–3 min of water adjustment. The group then gathers in the pool to review the swim skill of the week with time to practice with their interventionist. These skills include tolerating splashing, bubbles, submerging, floating, kicking, and scooping arms. The group is then dismissed to stations where each child and interventionist follow a visual schedule to rotate through 6 stations individually to address individual swim skills, which are adjusted to each child's level by the interventionist.

Each station reflects a different swim skill including bubbles/submersion, kicking, floating, jumping, throwing & scooping items, monkey crawl, and one for individual skills. The individual swim skill station allows children and their interventionist to work on additional skills not addressed by the stations that week or have additional practice on a skill with the leading therapist, a licensed occupational therapist most often the first author. Throughout the stations, therapeutic techniques included AquOTic active ingredients such as positive reinforcement, modeling, child-led and play-based activities, shaping motor movements, and repetition. The session ends with the group re-visiting that week's skill together and then singing songs or a group game to end the session.

#### 2.2.2 Training for interventionists

All interventionists underwent comprehensive training in AquOTic intervention fidelity before working with study participants. Training included both land and water-based education, focusing on each of the fidelity active ingredients and their application at each skill station. In addition, all interventionists had completed pediatric coursework, which included detailed information about autism as a diagnosis, grading and adapting, play as a therapeutic technique and other common intervention approaches.

Following each session, interventionists completed a self-report fidelity checklist to ensure adherence to the intervention protocol. Weekly feedback was provided by the lead therapist, and individualized support or additional training was offered as needed to ensure ongoing fidelity and professional development.

#### 2.2.3 Training for blinded assessors

All assessors were trained on the Water Orientation Test-Alyn (WOTA) and the Swim Skills Checklist (SSC). Assessors first read both assessments and reviewed the swim skills in each. The blinded assessors then determined how items could be grouped together across both assessments for efficiency of assessing and scoring. Each assessor completed in-water training where they administered the assessments to one neurotypical child and one child on the autism spectrum. Initial agreement for rating items was 90% or higher with the first author. Video recordings were obtained, and the third author, who was also the primary blinded assessor, verified scores as needed.

### 2.3 Outcome measures

#### 2.3.1 Water orientation test-Alyn (WOTA)

The WOTA measures a child's level of adjustment and functional ability in water. The evaluation is based on the Halliwick concept and measures mental adjustment, postural balance, and the ability to move and change position in the water (18). The WOTA is a reliable and valid standardized assessment tool for children with physical disabilities, consisting of two versions (WOTA1 and WOTA2). The WOTA1 has 13 specific swim skills scored on a scale of 1–4 with a minimal detectable change score of 4.5 (18). The swim skills assessed include mental adjustment (items 1, 5-–), breath control (items 4, 8), and function (items 2, 3, 9–13). Mental adjustment includes entering the water willingly, splashing, and side/back floating. Breath control includes being able to blow bubbles and submerge under water. Items related to function include standing or sitting in the water, entering/exiting the pool, and holding/moving across a rope. The WOTA2 has 27 specific swim skills scored on a 0–3 scale with a minimal detectable change score of 11.5 (18). The items assessed on the WOTA2 are broken into four sections: (A) mental adjustment (item 1), (B) breath control (items 2–6), and C/D) function (items 7–27). Mental adjustment on the WOTA2 assesses a child's adjustment to the pool environment. Breath control includes skills such as blowing bubbles through the mouth, and the nose, rhythmically moving and breathing, and alternating blowing bubbles from the mouth and nose. Section C on the WOTA2 includes items such as entering/exiting, walking, jumping, back float, prone float, changing orientation while floating, and submerging. Section D of the WOTA2 focuses on skills such as progression on the back, freestyle, backstroke, and breaststroke. Higher scores indicate more proficient swim skills. Across both measures, the inter-rater reliability has been reported as excellent (WOTA1 ICC = .97; WOTA2 ICC = .97) with reliability for individual items as fair to good (kappa >.4) (18). Our prior work (23) and those of others (17, 22) have shown that the WOTA is a sensitive measure to change in water competency among children on the autism spectrum.

#### 2.3.2 Swim skills checklist (SSC)

The SSC was developed by our research team to obtain additional information pertaining to water competency not assessed on the WOTA1 or WOTA2. The SSC was developed based on the guidance of the American Red Cross and other established checklists for water competence (25, 26). The SSC contains 14 items including kicking, treading water, jumping in the pool, and returning to the edge of the pool. Higher scores represent more swim skills.

#### 2.3.3 Daily documentation

Interventionists completed a structured daily documentation after each session noting any adverse events, specific goals for the child, and notes for the day including activities completed, favorite toys, and cues that the child responded to.

#### 2.3.4 Fidelity checklist

Interventionists completed a self-report fidelity checklist after each session noting the implementation of AquOTic active ingredients including visual modeling of techniques by the interventionist, use of verbal or other praise for attempts at skills, using a preferred toy or song to encourage the swimming motions, and repetition of skills and activities to reinforce motor learning. Please see Supplementary Material for the checklist and associated active ingredients.

### 2.4 Power analysis

Based on a recent meta-analysis of behavioral aquatic therapy for children on the autism spectrum (6–12 years) (20), and our pilot feasibility study with AquOTic (23), the effect size for pre- to post-therapy change in WOTA scores was large (Cohen's D = 1.65) (27). To determine the required sample size for our study, we performed a power analysis using G*Power software (version 3.1.9.7) for an *F*-test ANOVA repeated measures within-between factors analysis. The input parameters for G*Power were as follows: Effect size (f): 0.4; *α* error probability: 0.05, Power: 0.95, Number of groups: 2; number of measurements: 2. The minimal sample required was 24 participants per group. Thus, our sample of 37 participants (25 controls and 37 intervention) was sufficiently powered to achieve statistical significance.

### 2.5 Statistical analysis

Frequencies and percentages were used to describe the categorical characteristics of the sample, and medians and interquartile ranges (IQR) were used to describe continuous measures. Sample demographics and baseline scores were stratified by those who received the AquOTic intervention first and those who were randomized to the control group first. Average WOTA1, WOTA2, and SSC scores were provided at each time point using a repeated measures mixed effects model, where the time points were assumed to follow a first-order autoregressive [i.e., AR(1)] correlation structure. For those who had pre- and post-AquOTic intervention time points, the Cohen's D for overall scores and individual swim skills was calculated to determine the mean standardized difference before and after receiving the intervention. Based on the American Red Cross definition of water competency, we identified six skills (Entry, Total Submersion, Recovery to surface with 1 min of floating/treading, Change in body orientation, Propulsion for 25 yards, and Exit Water) from the WOTA and SCC to examine change in water competency. Significance was assessed at the 0.05 level, and Cohen's D greater than 0.80 were considered large effect sizes. All analyses were performed using SAS v9.4.

## 3 Results

### 3.1 Participant demographics

Among the 37 children who participated in this study, 12 were assigned to the AquOTic intervention first, and 25 were assigned to waitlist control groups (Table 2). The median age was six for both groups (AquOTic IQR: 5–7; Control IQR: 6–7), and the proportion of males was similar in each (AquOTic: 75.0%, *n* = 9; Control: 76.0%, *n* = 19). The waitlist control group had 6% more children who identified as non-white, and the intervention group had 12.7% more children who identified as Hispanic/Latino compared to the waitlist controls. The median number of sessions attended was nine for both groups (AquOTic IQR: 9–10; Control IQR: 8–10). Based on the interventionist fidelity checklist, all key ingredients were present >90% of the time across all sessions. No adverse events or injuries were reported among the children or the interventionists during AquOTic sessions.

**Table 2:** Sample demographics and scores on the water orientation test-Alyn (WOTA) 1 and 2, swim skills checklist, and attendance (*N* = 37).

	AquOTic first (*n* = 12)	Waitlist control^a^ (*n* = 25)
Demographics		
Age (in years), median (IQR)	6 (5, 7)	6 (6, 7)
Male, *n* (%)	9 (75.0%)	19 (76.0%)
Race, *n* (%)		
Asian	0 (0.0%)	4 (16.0%)
Black	2 (16.7%)	4 (16.0%)
White	6 (50.0%)	11 (44.0%)
More than 1 race	4 (33.3%)	6 (24.0%)
Hispanic/Latino, *n* (%)	2 (16.7%)	1 (4.0%)
WOTA 1 total score [median (IQR)]		
WOTA 1 total	36 (28, 42)	37 (30, 45)
WOTA 2 Scores [median (IQR)]		
Section A—mental adjustment	2 (2, 3)	3 (2, 3)
Section B—breathing control	1 (0, 5)	2 (0, 6)
Section C—functional goals	18 (11, 30)	20 (15, 29)
Section D—functional goals	0 (0, 0)	0 (0, 0)
Total	23 (14, 35)	24 (17, 35)
Swim Skills Checklist, median (IQR)		
Touches water	2 (1, 3)	2 (2, 3)
Breath control	0 (0, 1)	1 (0, 3)
Blows bubbles	3 (0, 5)	2 (1, 4)
Visual location & retrieval of item	0 (0, 0)	0 (0, 2)
Kicking leg motion	2 (2, 2)	2 (2, 2)
Kicking, arm position	2 (1, 2)	1 (0, 3)
Jumping in from side	3 (2, 3)	3 (1, 5)
Prone gliding	1 (0, 2)	1 (0, 1)
Tread water	0 (0, 0)	0 (0, 0)
Independent locomotion	0 (0, 0)	0 (0, 0)
Change directions while swimming	0 (0, 0)	0 (0, 0)
Rest breaks	0 (0, 0)	0 (0, 0)
Combined swim skills	0 (0, 0)	0 (0, 0)
Freestyle for 5 m (15 ft)	0 (0, 0)	0 (0, 0)
Total	12 (6, 19)	13 (11, 18)
Number of sessions attended, median (IQR)	9 (9, 10)	9 (8, 10)

^a^ 2/25 waitlist controls did not attend AquOTic intervention.

### 3.2 Change in WOTA 1 and 2 with AquOTic

Average WOTA1 scores increased significantly after participants received AquOTic (Δ = 5.7; 95% CI: 3.7–7.8; *p* < 0.001), and average WOTA2 scores increased significantly after participants received AquOTic (Δ = 9.0; 95% CI: 5.7–12.3; *p* < 0.001; See Table 3). While WOTA1 and 2 scores increased from the participant's first baseline assessment (control time 1) to post-intervention, scores only increased significantly after participants received AquOTic. Moderate-to-large mean standardized differences in scores were also observed after participants received AquOTic (WOTA1 Cohen's *D* = 0.87; WOTA2 Cohen's *D* = 0.63).

**Table 3:** Effect sizes and average WOTA and swim skills over time with corresponding 95% Cis.

	WOTA 1	WOTA 2	Swim Skills Checklist
	Average (95% CI)		
Control time 1	36.7 (33.6, 39.7)	25.6 (19.8, 31.4)	13.4 (9.8, 17.0)
Control time 2	37.8 (34.9, 40.8)	26.4 (20.8, 32.0)	15.0 (11.5, 18.5)
Pre-AquOTic	37.1 (34.2, 40.1)	27.8 (22.2, 33.4)	15.6 (12.1, 19.1)
Post-AquOTic	42.9 (40.1, 45.6)	36.8 (31.4, 42.1)	23.2 (20.0, 26.5)
	Effect size^a^		
Post-pre AquOTic	0.87	0.63	0.86

^a^ Mean standardized differences (Cohen's D) of scores post vs. pre-AquOTic intervention.

### 3.3 Change in swim skills with AquOTic

**Figure 2:**
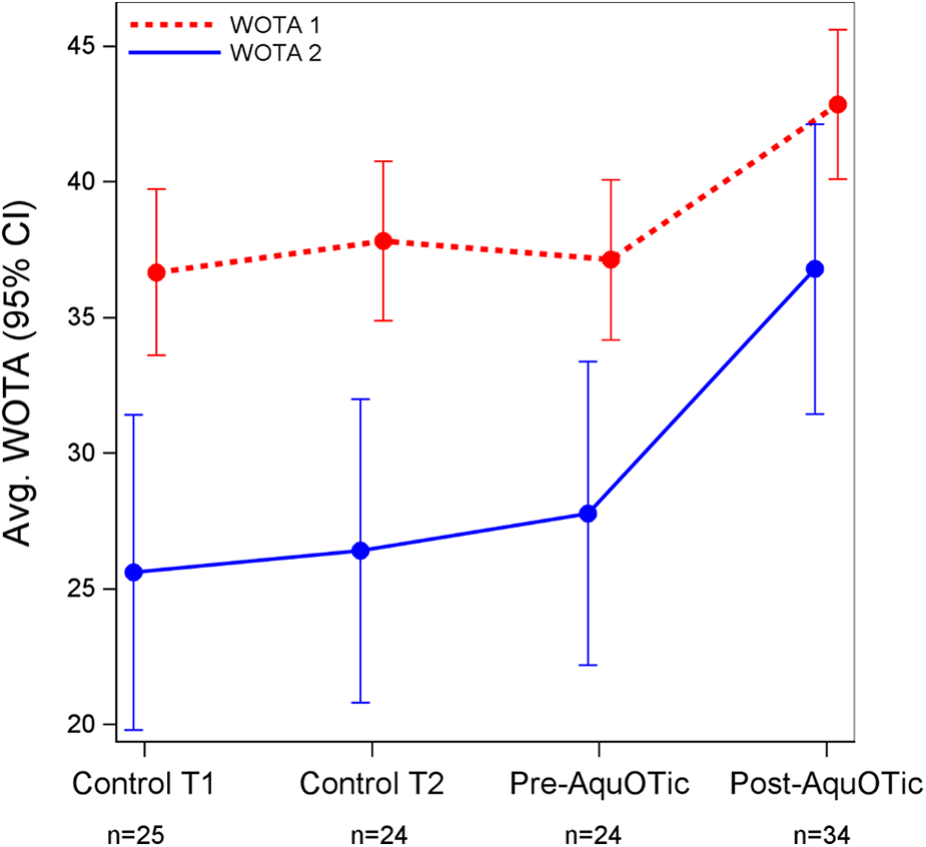
Average WOTA 1 and 2 over time with corresponding 95% CIs.

**Figure 3:**
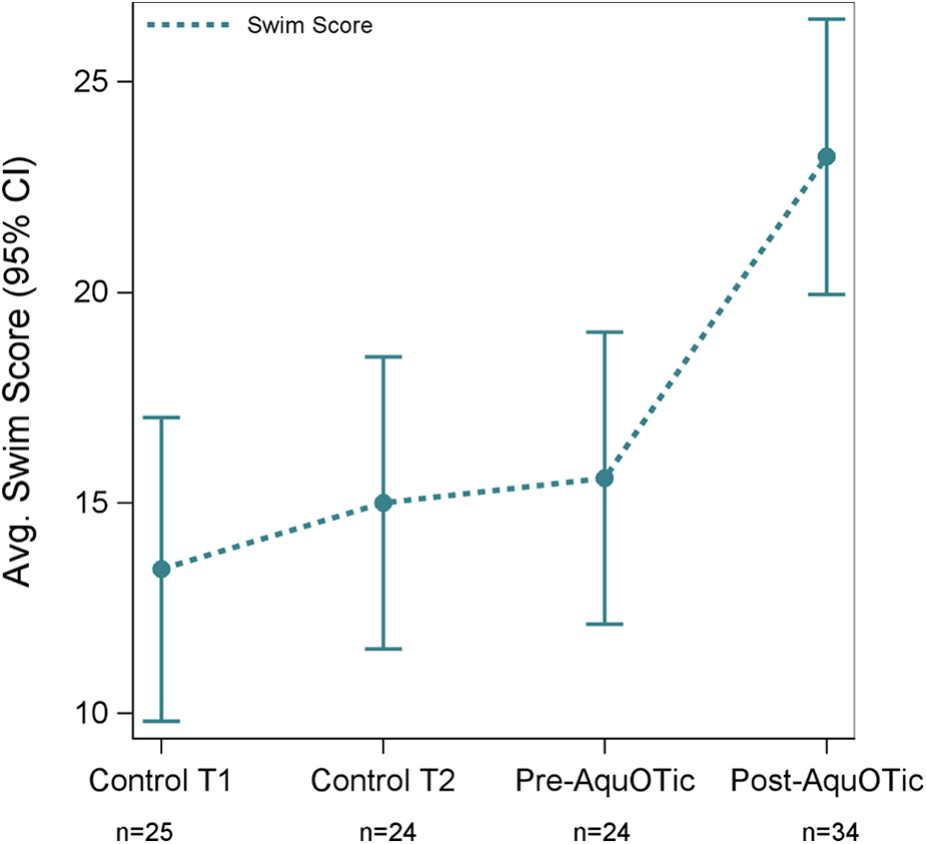
Average swim score based on the Swim Skills Checklist over time with corresponding 95% CIs.

Average swim skills based on the Swim Skills Checklist increased significantly after participants received AquOTic (Δ = 7.6; 95% CI: 5.3, 10.0; *p* < 0.001). While swim skills increased consistently over time, overall swim skill scores only increased significantly after participants received AquOTic. In addition, the effect size for swim skill score post- to pre-AquOTic intervention was large (Cohen's *D* = 0.86). Large effect sizes were observed in the following individual swim skills: touches water (Cohen's *D* = 0.93), breath control (Cohen's *D* = 0.90), visual location, and retrieval of item (Cohen's *D* = 1.08; Table 4). That is, the average child after AquOTic scored approximately 1 standard deviation above the average child before AquOTic (see Figures 2, 3).

**Table 4:** Mean standardized differences (Cohen's D) of individual swim skills post vs. pre-AquOTic intervention.

Swim Skills Checklist items	Cohen's D
Touches water	**0** **.** **93**
Breath control	**0**.**90**
Blows bubbles	0.53
Visual location & retrieval of item	**1**.**08**
Kicking leg motion	0.55
Kicking, arm position	0.42
Jumping in from side	0.53
Prone gliding	0.55
Tread water	0.64
Independent locomotion	0.41
Change directions while swimming	0.35
Rest breaks	0.44
Combined swim skills	0.53
Freestyle for 5 m (15 ft)	0.29

Bold values are considered to be large (>0.80) effect sizes.

### 3.4 Change in water competency with AquOTic

Using the participant's most recent baseline assessment prior to the intervention and their measurements post-AquOTic, children improved in most skill components of water competency (Table 5). Largest percent changes in water competency included total submersion (+40% improvement) and exiting the water (+22.9% improvement). Propulsion for 25 yards was the only category that did not show improvement (+0% change).

**Table 5:** Change in water competency from most recent baseline assessment to post-AquOTic intervention.

Skills	Baseline (*n* = 37)	Post-aquOTic (*n* = 34)
Entry^a^	28 (75.7%)	30 (88.2%)
Total submersion	9 (24.3%)	22 (64.7%)
Recovery to surface with 1 min of floating/treading	0 (0%)	1 (2.9%)
Change in body orientation (180^o^ rotation & face exit)	2 (5.4%)	5 (14.7%)
Propulsion for 25 yards^a^	0 (0.0%)	0 (0.0%)
Exit water^a^	22 (59.5%)	28 (82.4%)

^a^ Items were from WOTA 2. All remaining items were from the swim skills checklist.

## 4 Discussion

This is one of the first single-blind randomized controlled trials of a manualized water competency and safety intervention among children on the autism spectrum. Our results show that AquOTic results in significant improvements in elements of water competency and safety among children on the autism spectrum as measured by the standardized WOTA 1 and 2 measures. In addition, children receiving AquOTic showed substantial improvements in water competency skills as defined by the American Red Cross. Our findings align with recent reviews of swim intervention programs for children on the autism spectrum that show significant improvements in swim skills and water safety after at least 10 h of intervention (15, 17, 21). Our findings expand on our prior feasibility study with AquOTic which highlighted the safety, feasibility, and acceptability of the intervention (23). Noteworthy, trained assessors who were unfamiliar with the child were blind to group assignment and pre- vs. post-status, reducing bias.

According to the World Health Organization, basic swim skill intervention could prevent over 238,000 fatal and 549,000 nonfatal drowning cases worldwide from 2020 to 2050 (28). By investing in prevention, more than $400 billion in potential economic losses due to drowning can be avoided (28). Each dollar invested into drowning prevention can yield returns of up to nine times, promoting public health and well-being (28). Due to the disproportionate prevalence of drowning among children with autism, targeted swim skill intervention for this population is critical. Families of children on the autism spectrum have few resources for adaptive swim programs that are appropriate for their child and often must resort to private lessons, which may or may not include an instructor familiar with the needs of their child. AquOTic is a novel application of occupational therapy practice to water competency training for children on the autism spectrum, making it scalable and widely acceptable. Occupational therapy is one of the most requested and highly utilized interventions for autism (29). Occupational therapy practitioners should consider water competency skill development in their routine care, to mitigate drowning risks. The AquOTic program provides an innovative approach to serving families of children on the autism spectrum by offering a specialized aquatic program that could be implemented by widely available occupational therapy practitioners in their local communities.

A common concern across reviews synthesizing aquatic interventions among children on the autism spectrum and other neurodevelopmental conditions is the lack of consistent standardized outcome measures across studies. The variability in outcome measures used across studies hampers the ability to compare intervention types and consistently examine the effects of intervention intensity and frequency. The results from our study show that the WOTA 1 and 2 are sensitive to change and feasible to use among children on the autism spectrum. However, the WOTA 1 and 2 do not include all critical water competency skills as defined by the American Red Cross (7). Thus, there is a need for better standardized water competency measures that are tailored for children with neurodevelopmental conditions. Prior studies using the WOTA 1 and 2 have had sample sizes less than seven and did not use control groups (15, 22). The results from our study show that AquOTic effect sizes with WOTA 1 and 2 are large (Cohen's D WOTA 1 = .87; WOTA 2 = .63). A recent systematic review and meta-analysis of aquatic therapy among children with neurodevelopmental conditions used the Humphries’ Assessment of Aquatic Readiness (HAAR) checklist across 4 trials (20). The review showed that aquatic therapy improved mental adjustment, rotation, balance and control, and independent movement in water compared to land-based exercises with similar effect sizes as the current study (20). In the current study, children demonstrated the highest gains in skills related to mental adjustment, breath control, and visual location and retrieval of items. These findings are also consistent with prior occupational therapy-based swim interventions in children on the autism spectrum, where 8 h of intervention resulted in the highest gains in breath control, propulsion, and changing positions while swimming (30).

Gaining proficiency in water competency is a protracted process requiring multiple instructional sessions combining learning alongside developmental maturation (31). Our results and those from a systematic review of 23 studies of swim programs for individuals on the autism spectrum (15) indicate that at least 8–10 h of therapeutic swim instruction is required to teach children foundational swim and water safety skills. While participants in our study showed substantial improvement in water competency skills, they did not meet criteria for water competency. Although there is no defined cut-off for water competency on the WOTA2, the maximum score is 81. In this study, the mean post-AquOTic total WOTA2 score was 36.8 points, which clearly shows that children have the potential to continue learning water competency skills and highlights the need for higher doses of AquOTic. Current studies of swim interventions in autism show that children demonstrate the highest gains in water competency with 24–28 h of intervention (23, 30, 32, 33). Exposure to positive aquatic experiences and prior swim lessons has been associated with greater swim skills among children on the autism spectrum (34, 35). Thus, it is critical for families to have access to therapeutic aquatic intervention programs that support positive aquatic exposure. Future research is needed to examine the ideal dosage (number of swim intervention hours) to achieve water competency for children on the autism spectrum.

There is a need for scalable and community-based aquatic programs tailored to meet the needs of children on the autism spectrum and other neurodevelopmental conditions. AquOTic is a personnel-intensive program given the structure of 1:1 child: interventionist pairing along with a lead supervising therapist. In the current study, all interventionists were graduate-level occupational or physical therapy students. The advanced training among the study interventionists potentially contributed to the high intervention fidelity. Prior studies also note that skilled clinical therapists providing the intervention result in children on the autism spectrum gaining greater skills (30, 33). Future research should investigate optimal implementation strategies such as involving individuals with high-school or undergraduate-level training and developing water competency/safety instruction training for non-professional individuals to ensure scalability in the community.

### 4.1 Study limitations and important future directions

Although this study had several strengths, including a racially diverse sample, standardized measures with blind assessors, high fidelity, and low attrition, there remain some limitations. This study lacked an active control group of a traditional community-based swim intervention to use as a comparison with AquOTic. For ethical reasons, children in the waitlist control group were not asked to restrict exposure to other aquatic activities during their time as controls. Our study findings clearly show that children in the waitlist control group with multiple assessments showed significant gains in water competency only after AquOTic intervention. Moreover, 40% of caregivers of children on the autism spectrum (*n* = 15) reported being unable to find private or group swim lessons that met their child's unique needs. Additionally, 14% (*n* = 5) had to discontinue lessons due to safety concerns or requests from the facility. These findings highlight significant gaps in the availability and suitability of traditional swim lessons for children on the autism spectrum. There is a need for long-term follow-up evaluations to examine the retention and consistency of swim skills over time. The primary outcome measure in this study was swim skills and water safety as measured by the WOTA 1 and 2 and the Swim Skills Checklist. Future research with large samples should examine the impact of swim interventions on drowning risks. Given the growing evidence highlighting the impact of aquatic therapy on other health outcomes for children on the autism spectrum (20, 22), future research should include outcome measures targeting cognitive, motor, sensory, play, and social skills. Aquatic exercise for children on the autism spectrum has also been shown to improve inflammatory serum levels and sleep patterns (36). Future research should also focus on caregiver-centered goals including caregiver stress around aquatic exposure and quality of life. This study's participants included 40% minimally verbal children (*n* = 14) based on parent-report and therapist-observation. However, verbal skills, IQ, and level of autism severity were not assessed using standardized measures. The results of this study highlight that aquatic intervention is feasible and beneficial for children across the spectrum. Minimally verbal children are often excluded from research and there is a critical need to ensure that children across the autism spectrum are represented in intervention research.

## 5 Conclusions

Overall, the present single-blinded RCT design of AquOTic intervention provides strong support that 10 weeks of an occupational therapy-based aquatic intervention significantly improves water competency including swim skills and water safety among children on the autism spectrum. The highest gains were observed in basic swim skills such as mental adjustment, breath control, and visual retrieval of items in the water. These findings highlight the need for long-term aquatic interventions for children on the autism spectrum. Future research should examine the ideal dosage to achieve water competency and the impact of aquatic intervention on other health outcomes.

## Data Availability

The raw data supporting the conclusions of this article will be made available by the authors, without undue reservation.
