# Supplementary material for: Improving water competency among children on the autism spectrum: the AquOTic randomized controlled trial
Source: Front Pediatr. 2024 Oct 7;12:1473328. doi: 10.3389/fped.2024.1473328 (PMC11491375; doi:10.3389/fped.2024.1473328)
Supplement: Supplementary file 1 [file Datasheet1.pdf]

AquOTic™ Fidelity Self Checklist

Each swim buddy (interventionist) needs to indicate whether or not each of the following active ingredients were used in today's session with your child.

Interventionist name	______________________
Child's Name	______________________
Date	______________________
Did the child attend today?	◯ Yes<br>◯ No
Therapist uses child-led techniques including interests, choices or modifies activity to follow child's preference/direction	◯ Yes<br>◯ No
Example of child-led techniques including interests, choices or modifies activity to follow child's preference/direction	______________________
Therapists uses play-based games/activities to elicit motor skills	◯ Yes<br>◯ No
Example of Therapist uses play-based games/activities to elicit motor skills	______________________
Therapist models activities/skills/game	◯ Yes<br>◯ No
Example of Therapist models activities/skills/game	______________________
Therapists grades activities at stations when needed	◯ Yes<br>◯ No

Example of Therapists grades activities at stations when needed	____________________
Therapist uses shaping/successive approximation of motor skills	◯ Yes<br>◯ No
Example of Therapist uses shaping/successive approximation of motor skills	____________________
Therapists uses repetition of motor skills (5-10 rep/time at each station)	◯ Yes<br>◯ No
Example of Therapists uses repetition of motor skills (5-10 rep/time at each station)	____________________
Therapist uses visual schedule or icons	◯ Yes<br>◯ No
Example of Therapist uses visual schedule or icons	____________________
Therapist ensures child is engaged and attending to the activity	◯ Yes<br>◯ No
Example of Therapist ensures child is engaged and attending to the activity	____________________
Child-therapist interaction shows therapeutic relationship	◯ Yes<br>◯ No
Example of Child-therapist interaction shows therapeutic relationship	____________________
Therapist uses natural or scheduled reinforcements	◯ Yes<br>◯ No
Example of Therapist uses natural or scheduled reinforcements	____________________
Therapist uses water properties and games to reinforce tactile/proprioception input	◯ Yes<br>◯ No
Example of Therapist uses water properties and games to reinforce tactile/proprioception input	____________________

Therapists provides sensory accommodations including adaptive devices (ie goggles, fins) based on child's level of arousal and sensory preferences (quiet voice, less crowded area of pool)	◯ Yes<br>◯ No
Example of Therapists provides sensory accommodations including adaptive devices (ie goggles, fins) based on child's level of arousal and sensory preferences (quiet voice, less crowded area of pool)	__________________________________
Session 10: therapist provides information to caregiver through hands on training in the water	◯ Yes<br>◯ No
Example of Session 10: therapist provides information to caregiver through hands on training in the water	__________________________________
